# The Genetic Landscape of Sleep Disorders in Parkinson’s Disease

**DOI:** 10.3390/diagnostics14010106

**Published:** 2024-01-03

**Authors:** Kallirhoe Kalinderi, Vasileios Papaliagkas, Liana Fidani

**Affiliations:** 1 Laboratory of Medical Biology-Genetics, School of Medicine, Faculty of Health Sciences, Aristotle University of Thessaloniki, 54124 Thessaloniki, Greece; sfidani@auth.gr; 2 Department of Biomedical Sciences, School of Health Sciences, International Hellenic University, 57400 Thessaloniki, Greece; vpapaliagkas@gmail.com

**Keywords:** Parkinson’s disease, sleep disorders, GBA, Parkin

## Abstract

Parknson’s disease (PD) is the second most common neurodegenerative disease, affecting 1% of people aged over 60. PD is characterized by a wide range of motor symptoms, however the clinical spectrum of PD covers a wide range of non-motor symptoms, as well. Sleep disorders are among the most common non-motor symptoms of PD, can occur at any stage of the disease and significantly affect quality of life. These include rapid eye movement sleep behavior disorder (RBD), restless legs syndrome (RLS), excessive daytime sleepiness (EDS), insomnia, obstructive sleep apnea (OSA) and circadian rhythm disturbances. One of the main challenges in PD research is identifying individuals during the prodromal phase of the disease. Combining genetic and prodromal data may aid the early identification of individuals susceptible to PD. This review highlights current data regarding the genetic component of sleep disorders in PD patients, focusing on genes that have currently been associated with this PD co-morbidity.

## 1. Introduction

Parkinson’s disease (PD) is the second most common neurodegenerative disease, after Alzheimer’s disease. It affects 1% of people aged over 60 and 3% of those older than 80 years [1]. The pathological hallmarks of the disease are the loss of the dopaminergic neurons in the substantia nigra, causing dopamine depletion in the striatum, and the presence of Lewy bodies (LBs) in the remaining neurons. PD is characterized by a wide range of motor symptoms, such as resting tremor, rigidity, bradykinesia, postural instability and freezing episodes. However, the clinical spectrum of PD is more extensive, covering a wide range of nonmotor symptoms, including cognitive and behavioral symptoms, sleep disorders, autonomic symptoms, sensory symptoms and fatigue [2]. Destruction of dopaminergic neurons precedes the onset of motor symptoms by many years and usually non-motor symptoms of PD occur earlier compared to motor symptoms. Current research is focused on the recognition of biological markers that could identify the disease in early stages, allowing early diagnosis and treatment of PD.

Sleep disorders, such as REM sleep behavior disorder (RBD), restless legs syndrome (RLS), excessive daytime sleepiness (EDS), insomnia, obstructive sleep apnea and circadian rhythm disturbances are common in idiopathic PD (iPD). In particular, individuals suffering from RBD carry a risk of >85% to manifest PD after 15–20 years, and the associated neurodegenerative process is α-synucleinopathy in 95% of cases [3]. The contribution of common genetic variants to the clinical variability in sporadic PD is a hot topic of scientific research. This review highlights current data regarding the genetic component of sleep disorders in PD patients, focusing on genes that have currently been associated with this PD co-morbidity.

For this narrative review article, we searched PubMed and Scopus databases for peer-reviewed research, review articles and meta-analyses regarding the role of genetics in sleep disorders in PD patients, published in the English language with no time restrictions. We also screened the references of the selected articles for possible additional articles in order to include most of the key recent evidence. Our research was conducted between November 2022 and November 2023. We used the terms: “Parkinson’s disease”, “rapid eye movement sleep behavior disorder”, “restless legs syndrome”, “excessive daytime sleepiness”, “insomnia”, “obstructive sleep apnea”, “circadian rhythm disturbances”, “gene”, “genetic”, “gene polymorphisms”, “SNP”, “genotype”, “allele”, “mutation”, “variant”, “familial PD”, “sporadic PD”, “autosomal dominant PD”, “autosomal recessive PD”, “risk factor”, “genetic susceptibility”, “prodromal”, “mutation carriers”, in various combinations. 

**Scheme 1:**
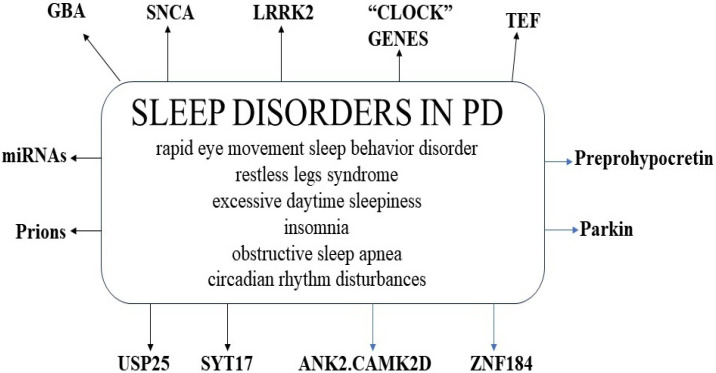
Genes and sleep disorders in PD.

Scheme 1 highlights the importance of the genes involved in sleep disorders and PD. 

## 2. Genes Involved in PD Patients’ Sleep Disorders

### 2.1. GBA

*GBA* is located on chromosome 1 (1q21) and encodes for the lysosomal enzyme glucocerebrosidase (GCase). GCase catalyzes the hydrolysis of glucocerebroside into glucose and ceramide. Mutations of *GBA* are associated with Gaucher’s disease (GD), a recessive lysosomal storage disorder that is characterized from reduced GCase activity and accumulation of glucocerebroside in the liver, spleen and bone marrow. Interestingly, it has been observed that both homozygote and heterozygote carriers of *GBA* mutations are at increased risk for developing PD. More specifically, the risk is significantly higher among those who are carriers of severe mutations compare to mild mutation carriers, with up to 10-fold and 2–3 fold increased risk for developing PD, respectively [4]. *GBA* variants are classified into severe variants (such as L444P, W291X, H225Q and IVS2 + 1G > A) and mild variants (such as N370S). In addition, several nonpathogenic variants of *GBA* in GD, such as E326K and T369M, have been found to increase the risk of PD. PD patients with severe variants of *GBA* have a younger age of onset, faster progression and more severe cognitive impairment than those with mild variants. *GBA* mutations are present in about 2–30% of PD patients, and carrier frequency can be very different across different populations [5]. 

RBD is a sleep disorder characterized by abnormal motor behavior associated with dream mentation and loss of atonia during REM sleep [3]. Scientific attention over RBD has increased due to its association with α-synucleinopathies. More specifically, there is accumulating evidence that the idiopathic/isolated form of RBD (IRBD) constitutes the prodromal stage of the α-synucleinopathies, as most of these cases are eventually diagnosed with PD or dementia with Lewy bodies (DLB), with an estimated rate of conversion of 34% after five years from the IRBD diagnosis, 74% after ten and 91% after fourteen years. Additionally, postmortem neuropathological studies in subjects initially diagnosed with IRBD showed widespread LBs and neurites containing α-synuclein as their main component [6]. RBD has been associated with *GBA* mutations both in GD patients and in PD patients. Gan-Or et al. found that *GBA* mutation carriers had an OR of 6.24 for RBD, and among PD patients, the OR for mutation carriers to have probable RBD (pRBD) was 3.13 [7]. Jesus et al. also found a higher prevalence of RBD in both deleterious *GBA*-carriers and benign *GBA*-carriers [8]. A “dose effect” of *GBA* mutations on PD phenotype was also described by Thaler et al., with a more severe PD phenotype in patients with GD and PD (GD-PD) as compared to *GBA*-related PD patients (GBA-PD) and iPD. These patients, in addition to an earlier age of onset, more severe motor impairment, poorer cognition and lower olfactory scores, also had a higher prevalence of RBD and higher frequencies of hallucinations compared to both GBA-PD and iPD [9]. Additionally, a recent study, confirmed that non-motor symptoms and a more severe and rapidly progressive disease course are seen more frequently among *GBA*PD patients in comparison to iPD. A higher occurrence of diurnal sleepiness was also observed in the carriers’ group [10]. The fact that the severity of the *GBA* mutation would correspond to the PD phenotype was also addressed in the study of Thaler et al. [11]. A total of 355 PD patients were included in this study; 152 iPD patients, 139 mild GBA (mGBA) mutation carriers, 48 severe GBA (sGBA) mutation carriers and 16 patients with GD and PD (GD-PD). Both sGBA and GD-PD had higher frequencies of RBD and hallucinations compared to the other groups of patients. Motor, cognitive, olfactory and psychiatric symptoms were also more severe in sGBA and GD-PD compared to mGBA and iPD, thus the severity of the PD phenotype is associated with the severity of the mutation in the GBA gene [11]. A recent meta-analysis study showed that PD patients with heterozygous *GBA* variants are at high risk of developing RBD. PD patients with *GBA* variants, like N370S and L444P, are at an even higher RBD risk than PD patients without these variants [3,12]. Moreover, in a recent study Perez-Lloret et al. found that *GBA*_N370S_rs76763715 was associated with more frequent pRBD among PD patients. In addition, some *GBA* variants, like rs2230288/E326K, rs75548401/T369M, and rs369068553/V460L, were also associated with more frequent non-motor symptoms, including RBD, as well as with a more aggressive motor disease [13].

### 2.2. SNCA

The α-synuclein (*SNCA*) gene has been mapped to human chromosome 4q21.3-q22 and encodes for α-synuclein, a 140 amino acid soluble protein expressed primarily in neural tissue and especially in presynaptic terminals. α-synuclein was the first gene implicated in the genetic pathogenesis of PD, especially with autosomal dominant PD. The A53T missense mutation was first identified in a large Italian–American family and in a number of Greek families [2]. Interestingly, increased expression of α-synuclein has been found to reduce the age of PD onset and increase the disease severity. α-synuclein in its native state is a soluble and unfolded protein. Due to its central hydrophobic region α-synuclein has an inclination to aggregate. It initially forms an intermediate structure called an oligomer or protofibril and in succession insoluble polymers or fibrils. These insoluble fibrils are the major component of LBs [14]. The regulation of the levels of monomeric and/or oligomeric α-synuclein in neurons appears to be critical; this regulation might be altered in the common PD forms due to mutations in the α-syn gene that can lead to increased α-synuclein expression, decreased clearance or both [15,16].

Toffoli et al., examined 113 consecutive patients with a diagnosis of iRBD (56 patients) or PD (with or without RBD, 57 patients). The *SNCA* 3′UTR regulatory region, which is involved in gene expression, was sequenced and the *SNCA* rs356165, rs3857053, rs1045722 polymorphisms were found to be more frequent in PD patients compared to iRBD patients. Different regulating sequences are present in the 3′UTR, such as those bound by microRNAs (miRNAs); thus, genetic variants in this region can affect miRNA activity, and alter the process of transcription, which ultimately affects protein production [17]. In another study, *SNCA* variants conferring risk of PD have also been found to predispose to an RBD phenotype and RBD has been proposed as a relevant marker for distinct PD subtypes. More specifically, pRBD was significantly associated with a known GWAS hit variant in the 5′ region of *SNCA*, highlighting how distinctive clinical features in PD may depend on the genetic basis of disease [18]. In a recent study, RBD and other sleep abnormalities were examined in carriers of the p.A53T *SNCA* mutation, using both subjective and objective measures, such as questionnaires and polysomnography (PSG) recordings. Sleep disorders were very common in A53T-PD carriers. More specifically, RBD was highly represented and was associated with hyposmia and cognitive decline. The increased presence of RBD may correlate with the high incidence of cognitive dysfunction in this group. Thus, impaired olfaction and RBD seem to evolve in parallel with the motor symptoms of the disease [19]. In a previous study, hyposmia was associated with RBD in PD patients with the minor G allele of rs894278, thus PD patients harboring this allele were more likely to have hyposmia and RBD simultaneously and worse disease progression, highlighting that some genotypic characteristics could participate in the co-occurrence of hyposmia and RBD in PD patients [20]. Clinical–pathological studies have also suggested that both hyposmia and RBD may be related to α-synuclein deposition [21]. Moreover, Lahut et al. examined a large *PARK4* PD pedigree and proposed that the risk for a future manifestation of PD is reflected in the global transcriptome of blood. More specifically, subtle complexin 1 (CPLX1) down-regulations were shown to distinguish prodromal PD cohorts (presymptomatic PARK4 heterozygotes and RBD individuals) from controls, suggesting that CPLX1 loss of function may act as a biomarker and modifier gene of PD risk [22].

### 2.3. LRRK2

The leucine-rich repeat kinase 2 (*LRRK2*) gene is located on chromosome 12q12. *LRRK2* variants have been recognized as the most common cause of both sporadic and familial PD to date. The available data suggest that the prevalence of the LRRK2 mutation varies markedly across populations. The most prevalent LRRK2 amino acid substitution, G2019S, is responsible for ~40% of familial and sporadic PD in Arab samples from North Africa, ~30% of familial PD in Ashkenazi Jewish populations, up to 6% of familial cases in Europe and up to 3% of apparently sporadic PD in Europe and North America [2,23]. The polymorphic variant G2385R has been strongly correlated to PD in the Chinese population. This variant is observed in 11.9% of PD patient, but is absent in Caucasian or Jewish patients [24]. *LRRK2* mutations have been associated with autosomal dominant PD and the clinical presentation is compatible with typical sporadic PD. However, the penetrance of *LRRK2* mutations is incomplete and age dependent, thus a proportion of asymptomatic individuals carrying mutations can be at the premotor stage of LRRK2-PD. The pathological findings in patients with *LRRK2* mutations are diverse comprising LB pathology and tauopathy with neurofibrillary tangles [24]. This diversity in pathology strongly suggests that LRRK2 is involved in multiple cellular processes and may be a central component of multiple signalling pathways that are crucial for proper functioning of neurons.

Sleep complaints have been found to be frequent in LRRK2-PD patients; in the study of Pont-Sunyer et al., 78% of them reported poor sleep quality, 33% sleep onset insomnia, 56% sleep fragmentation and 39% early awakening. Compared to iPD more frequent sleep onset insomnia, similar EDS and less prominent RBD were observed in LRRK2 PD patients. RBD and EDS seem not to occur in the premotor stage of LRRK2-PD. Compared to iPD, sleep onset insomnia was more frequent in LRRK2-PD and it was associated with depressive symptoms and poor sleep quality. Moreover, the fact that RBD in LRRK2-PD was less frequent could suggest that damage to the brainstem structures that regulate REM sleep atonia is less prominent in LRRK2-PD compared to IPD patients. This might be attributed to the heterogeneous neuropathological substrate found in LRRK2-PD [25]. In an observational cross sectional multi-center study, 253 subjects were enrolled and standard questionnaires assessing anxiety, depression, cognition, smell, non-motor symptoms and RBD were administered. On the RBD questionnaire of G2019S *LRRK2* mutation carriers, slightly more sleep problems were observed [26]. In the Chinese population, G2385R variant carriers had higher RBD screening questionnaire scores and more RBD symptoms compared with non-carriers. As RBD has been associated with a non-tremor predominant phenotype, and a higher rate of motor complications in PD patients, a relationship between RBD and motor phenotypes in PD patients with *LRRK2* variants may exist [27]. A case of a G2019S mutation carrier, affected by RLS with a positive family history of PD has also been reported. RLS is a stressful sensorimotor disorder characterized by an unpleasant urge to move the legs, that is worsened in the evening or the night, by resting or inactivity, e.g., lying or sitting, and is partially or totally relieved by movement. The prevalence rate of RLS is 5–15% in the general population. Most studies have shown a higher rate of RLS among PD patients compared to a control population [3]. The exact pathogenesis of RLS is not well understood, but the beneficial effects of dopaminergic drugs and the results of several functional imaging studies support the hypothesis of a dopaminergic nigrostriatal pathway involvement. RLS may be part of the spectrum of the clinical phenotypes related to the dopaminergic dysfunction caused by G2019S *LRRK2* mutation [28].

### 2.4. “CLOCK” GENES

The biological rhythm of the human body is regulated by the circadian system. Sleep disorders are related to alterations of the circadian rhythm, which is a physiological and behavioral cycle regulated by the suprachiasmatic nucleus (SCN) [29]. The circadian rhythm is executed by the interaction network of various circadian rhythm genes in almost all organs and tissues. The, so called, “clock” genes that regulate the circadian rhythm have recently attracted a lot of scientific attention [30]. Among these genes is, the circadian locomotor output cycle Kaput (*CLOCK*) gene that regulates the circadian rhythm in the brain and periphery and can modulate dopamine synthesis [31]. Importantly, *CLOCK* can regulate the transcription of tyrosine hydroxylase [32], which is an enzyme implicated in dopamine synthesis. After adjusting for other associated factors that may affect sleep, including depression, severity of motor symptoms, age, sex, and disease duration, in the Chinese population, the *CLOCK* 3111T/C variant carriers were found to be more susceptible to the development of sleep disorders. Thus, the *CLOCK* 3111T/C polymorphism could be an independent risk factor for sleep disorders in PD. Moreover, the proportion of patients with early onset PD in the carrier group was significantly higher than that in the noncarrier group, suggesting that the *CLOCK* 3111T/C variant may lower the PD age of onset [33].

The levels of expression of two other “clock” genes that regulate the circadian system, namely brain and muscle Arnt-like protein 1 (BMAL1) and period 1 (PER1), have also been examined through administration of melatonin (MEL). MEL is a hormone produced by the pineal gland that regulates the sleep-wake cycle and at pharmacological doses it is used to reduce sleep disorders. In rats, the administration of rotenone shows a decreased expression of the clock genes that are restored by administering MEL [34]. Interestingly, in the study of Delgado-Lara et al., MEL was administered to PD patients and was found to increase the BMAL1 levels, without affecting PER1 levels [35]. Moreover, several studies have found abnormal expression of circadian rhythm genes in peripheral blood lymphocytes (PBLs) from PD patients. Bmal1 and brain and muscle Arnt-like protein 2 (Bmal2) have been found significantly decreased in PD, while Bmal1 levels have been positively correlated with PD severity and sleep quality [36,37]. In another study, the expression levels of “clock” genes *BMAL1*, *CLOCK*, cryptochrome 1 (*CRY1*), *PER1* and period 2 (*PER2*) were significantly decreased in the peripheral blood mononuclear cells (PBMCs) of PD as compared to controls. Plasma MEL levels were also decreased in PD patients compared to controls. Based on these results, BMAL1, CLOCK, CRY1, PER1, PER2 and MEL have been proposed as potential biomarkers for evaluating the sleep-wake rhythm disturbances in PD patients [38,39]. Ghrelin, an orexigenic hormone, has also been found to have a strong endogenous circadian effect. Ghrelin concentration varies, with a peak in the day and a trough at night. Its level also depends on food intake [40]. Circulating ghrelin may act on the circadian system as a potential feedback signal for the SCN. Importantly, the genetic deletion Bmal1 abolishes the circadian rhythmicity of ghrelin signaling and affects ghrelin mRNA production. Thus, ghrelin may be linked with “clock” genes expression [41]. Interestingly, the therapeutic potential of mesenchymal stem cell-derived exosomes (MSC-EXOs) on sleep disorder in a 6-hydroxydopamine model of PD rats was recently investigated and MSC-EXOs were found to improve circadian rhythm in PD. More specifically, BMSC-derived exosomes rich in Wnt5 were effective in treating PD-related circadian rhythm dysfunction via enhanced peroxisome proliferator-activated receptor gamma (PPARγ) activity [42]. PPARγ is a member of the steroid hormone receptor superfamily. It is distributed throughout the basal ganglia and is co-expressed with dopamine receptors at local brain sites. Interestingly, treatment of PD rats with BMSC quiescent-EXOs and BMSC induced-EXOs significantly reversed the effects of 6-OHDA on SCN expression of CLOCK, Bmal and Per2 [42].

### 2.5. TEF

The thyrotroph embryonic factor (*TEF*) gene is located on chromosome 22q13. TEF is a transcription factor of the proline and acidic amino acid-rich basic leucine zipper (PAR bZip) family, which plays a major role in circadian rhythm regulation [43]. Circadian dysfunction may be a key cause of non-motor symptoms, especially for premotor symptoms, like sleep disturbances. The TT genotype of rs738499 *TEF* polymorphism has been associated with rapid deterioration in sleep quality in PD patients. Importantly, PD patients with more severe sleep disturbance at baseline presented a faster deterioration rate of sleep quality [43,44]. *TEF* is one of the downstream genes in the circadian gene network. It is expressed in various cells and tissues, and could be used as a predictive marker of the progression of sleep disturbances in PD patients. Notably, TEF may regulate other neurotransmitters, like dopamine and serotonin [45].

### 2.6. Preprohypocretin

A possible link between the dopaminergic and hypocretinergic system with respect to sleep-wake regulation has been suggested. Hypocretins play a major role in the pathophysiology of narcolepsy [46]. Narcoleptic patients suffer from symptoms such as EDS and shortened latency of sleep. In patients with PD, hypocretin levels were negatively correlated with disease severity. Dopamine is believed to play a crucial role in sleep regulation. Animal and postmortem studies in humans have shown that the dopaminergic system is disturbed in narcolepsy [47]. The D2-receptor binding has been also found elevated in narcolepsy and has been associated with the frequency of sleep attacks [48]. Moreover, the level of COMT activity, one of the major enzymes in dopamine metabolism, has been disease implicated in narcolepsy severity [49]. Hypocretin-1 and hypocretin-2 are neuropeptides processed from a common precursor, preprohypocretin [46]. Ιnterestingly, in the study of Rissling et al., the T allele of the -909T/C preprohypocretin polymorphism was associated with a significantly increased risk of developing sudden onset of sleep in PD patients [50].

### 2.7. Parkin

Parkin is one of the largest genes in the genome mapped to human chromosome 6q25.2-27. Parkin mutations have been found in PD patients of different ethnicity and have been considered as the major mutant factor for familial autosomal recessive juvenile parkinsonism (ARJP), with 50% of ARJP beyond the age of 25 and 3–7% of PD patients at ages of 30–45 years, carrying a mutation in this gene [2,51]. Until now, more than 100 different mutations have been identified, including deletions, insertions, duplications, triplications and point mutations [52,53]. Interestingly, in most PD cases with parkin mutations LB pathology is absent, however exceptions have been observed [54]. Interestingly, in 10 patients with *parkin* mutation, RBD was frequently observed, suggesting that mechanisms other than synuclein deposition can cause RBD in PD patients [55]. Similarly, in another study in 11 consecutive patients with two *parkin* mutations, their sleep phenotype was similar to that in iPD, except that RLS was more prevalent and secondary narcolepsy was absent [56]. Thus, the α-asynuclein deposit appears not to be mandatory for developing RBD in neurodegenerative diseases, such as PD. In a systematic review and meta-analysis, no correlation between *parkin* variants and the risk and severity of sleep disturbances in PD patients was found [12]. Additional studies examining sleep disorders in patients with *parkin* mutations are needed.

### 2.8. ZNF184

The zinc finger protein 184 (*ZNF184*) is located on chromosome 6 and encodes a Kruppel C2H2-type zinc-finger protein family member, which participates in gene expression regulation [57]. The genetic basis of pRBD in PD was recently examined and the *ZNF184*_rs9468199 polymorphism was found to increase the risk of pRBD [13]. This variant was previously associated with more frequent RBD in the Chinese population [58]. Further studies are needed to elucidate the exact role of *ZNF184* in RBD in PD.

### 2.9. ANK2.CAMK2D

Ankyrin 2 (*ANK2*) is located on chromosome 4 and encodes a member of the ankyrin family of proteins that link the integral membrane proteins to the underlying spectrin-actin cytoskeleton Ca^2+^/calmodulin-dependent protein kinase II (*CAMK2*). It is located on chromosome 5 and the product of this gene belongs to the serine/threonine protein kinases family, and to the Ca^2+^calmodulin-dependent protein kinases subfamily [59]. *CAMK2* is a key player in synaptic plasticity and memory formation. Interestingly, Camk2b knockout mice show reduced sleep, whereas the phosphorylation states of CaMK2 appear to control sleep induction and maintenance processes differently [60]. The *ANK2.CAMK2D*_rs78738012 polymorphism was recently found to increase the risk of pRBD [13].

### 2.10. SYT17

The synaptotagmin 17 (*SYT17*) gene is located on chromosome 16. SYT17 has calcium ion binding, phospholipid binding and syntaxin binding activity [61]. SYT17 is believed to be implicated in regulating circadian rhythms. The suprachiasmatic nucleus has been found to express high levels of SYT17 [62]. Interestingly, in the study of Perez-Lloret, the *COQ7.SYT17*_rs11343 polymorphism was found to reduce pRBD risk [13]. Further studies are required to confirm these findings.

### 2.11. USP25

The Ubiquitin Specific Peptidase 25 (*USP25*) gene is located on chromosome 21. USP25 has been found to have a crucial role in innate immunity [63] and to regulate microglial homeostasis and neuroinflammation during neurodegeneration [64]. In a small population study, Gan-Or et al. indicated that the *USP25* rs2823357 polymorphism was associated with faster progression to synucleinopathy from RBD. More specifically, homozygous carriers of the *USP25* rs2823357 polymorphsim progressed to synucleinopathies faster than others [65]. Notably, PD is a well-known synucleinopathy; *USP25* was previously implicated in a GWAS PD study [66], however this finding was not further replicated [67].

### 2.12. miRNAs

MicroRNAs (miRNAs) are coded in genes. They are expressed, and ultimately processed through a multi-step pathway. miRNAs are short double-stranded ribonucleotides that down-regulate mRNAs by RNA interference (RNAi). Dysregulation of miRNAS has been reported in PD [68]. Interestingly, dysregulation of the miR-19b has been previously observed in the prodromal stage of synucleinopathies. More specifically miRNAs were examined in patients with iRBD who converted to either PD or Dementia with Lewy bodies (DLB) compared to those who remained disease-free. miR-19b was down-regulated almost five years before the diagnosis of overt synucleinopathy, suggesting its possible relevance as a predictive biomarker for PD or DLB [69]. Interestingly, in a recent study that investigated possible biomarkers in the blood of patients at risk of developing PD, at the prodromal stage the expressions of 19a, 19b and 29a miRNAs decreased in plasma in those patients at risk of developing PD [70]. Additional studies are needed to determine whether miRNAs can be considered as useful prodromal disease progression markers for PD/DLB.

### 2.13. Prions

Prion protein (PrP) is a membrane-binding glycoprotein which is involved in molecular pathways that are crucial for neuronal differentiation, synaptic development, cellular oxidative stress response and cell–cell adhesion. Due to changes in its structure, PrP forms an infectious pattern called PrPSc, and has been associated with various diseases including bovine spongiform encephalopathy, scrapie and Creutzfeldt–Jakob disease [71]. Importantly, *cerebrospinal fluid* (CSF) PrP levels have been found to be altered in various neurodegenerative diseases [72]. Zhang et al., examined the correlation between the CSF PrP level and sleeping behavior in PD. Patients with PD complicated with RBD had significantly elevated CSF PrP mRNA and protein levels compared to both PD patients without sleeping disorder and healthy individuals [73]. The validation of PrP CSF biomarker to predict the development of PD in patients with iRBD is still in its infancy. Additional studies need be carried out in patients with iRBD in order to define the role of CSF biomarkers as predictors of this conversion.

## 3. Conclusions

Over the last few years, there has been a major shift in investigating the genetic background of specific PD subtypes, focusing especially on non-motor symptoms that most often precede the diagnosis of PD [45]. Sleep disorders, such as RBD, RLS, EDS and insomnia, are the most common non-motor feature of PD and often antedate PD, suggesting that sleep disorders are closely related to PD pathophysiology. One of the main challenges in PD research is identifying individuals during the prodromal phase of the disease. Combining genetic and prodromal data may aid the identification of individuals susceptible to PD. Currently, a number of genes have been associated with sleep disorders in PD, however additional studies on the genetics of this PD co-morbidity are of great importance, as genetic stratification of patients may substantially contribute to the early recognition of individuals at risk for PD (Table 1). The mapping the genetic landscape of PD will lead to major advances in our understanding of the pathophysiology of this movement disorder and is anticipated to have important implications for future PD therapeutic strategies.

## Figures and Tables

**Table 1:** Main genetic data regarding sleep disorders in PD.

Gene	Genetic Data	References
**GBA**	*GBA* mutations carriers—OR = 3.13 for pRBD sGBA and GD-PD had higher frequencies of RBD A recent meta-analysis PD heterozygous *GBA* patients—high risk RBD *GBA* variants, like N370S and L444P- higher RBD risk in PD patients *GBA*_N370S_rs76763715—more frequent pRBD among PD patients *GBA* variants, like rs2230288/E326K, rs75548401/T369M, and rs369068553/V460L—RBD	[3,7,11,12,13]
**SNCA**	*SNCA* variants conferring risk of PD—increase risk of pRBD a marker of PD subtypes Sleep disorders like RBD—more common in A53T-PD carriers PD patients with minor G allele of rs894278—more likely to have hyposmia and RBD simultaneously and worse PD progression	[18,19,20]
**LRRK2**	Sleep complaints RBD, and RLS have been found to be frequent in LRRK2-PD patients Relationship between RBD and motor phenotypes in *LRRK2* PD patients	[25,26,27,28]
**“CLOCK” GENES**	the *CLOCK* 3111T/C polymorphism could be an independent risk factor for sleep disorders in PD *CLOCK* 3111T/C variant may lower the PD age of onset Bmal1 levels have been positively correlated with PD severity and sleep quality	[33,36,37]
**TEF**	TT genotype of *TEF* rs738499—rapid deterioration in sleep quality in PD	[43,44]
**Preprohypocretin**	T allele of preprohypocretin -909T/C—sudden onset of sleep in PD	[50]
**Parkin**	PD with *parkin* mutation-frequent RBD, RLS	[55,56]
**ZNF184**	*ZNF184*_rs9468199—increase the risk of pRBD	[13,58]
**ANK2.CAMK2D**	*ANK2.CAMK2D*_rs78738012—increase the risk of pRBD	[13]
**SYT17**	*COQ7.SYT17*_rs11343—reduce pRBD risk	[13]
**USP25**	homozygous carriers of *USP25* rs2823357—faster progression to synucleinopathies	[65]
**miRNAs**	miR-19b was downregulated ~5 years before the diagnosis of overt synucleinopathy	[69]
**Prions**	PD complicated with RBD—elevated CSF PrP mRNA and protein levels	[72]
